# Acacia honey accelerates in vitro corneal ulcer wound healing model

**DOI:** 10.1186/s12906-016-1248-0

**Published:** 2016-07-29

**Authors:** Norzana Abd Ghafar, Choy Ker-Woon, Chua Kien Hui, Yasmin Anum Mohd Yusof, Wan Zurinah Wan Ngah

**Affiliations:** 1 Department of Anatomy, Faculty of Medicine, Universiti Kebangsaan Malaysia Medical Centre (UKMMC), Jalan Yaacob Latif, Bandar Tun Razak, Cheras, 56000 Kuala Lumpur Malaysia; 2 Department of Physiology, Faculty of Medicine, Universiti Kebangsaan Malaysia Medical Centre (UKMMC), Jalan Yaacob Latif, Bandar Tun Razak, Cheras, 56000 Kuala Lumpur Malaysia; 3 Department of Biochemistry, Faculty of Medicine, Universiti Kebangsaan Malaysia Medical Centre (UKMMC), Jalan Yaacob Latif, Bandar Tun Razak, Cheras, 56000 Kuala Lumpur Malaysia

**Keywords:** Acacia honey, Corneal fibroblasts, In vitro corneal ulcer, Wound healing

## Abstract

**Background:**

The study aimed to evaluate the effects of Acacia honey (AH) on the migration, differentiation and healing properties of the cultured rabbit corneal fibroblasts.

**Methods:**

Stromal derived corneal fibroblasts from New Zealand White rabbit (*n* = 6) were isolated and cultured until passage 1. In vitro corneal ulcer was created using a 4 mm corneal trephine onto confluent cultures and treated with basal medium (FD), medium containing serum (FDS), with and without 0.025 % AH. Wound areas were recorded at day 0, 3 and 6 post wound creation. Genes and proteins associated with wound healing and differentiation such as aldehyde dehydrogenase (ALDH), vimentin, alpha-smooth muscle actin (α-SMA), collagen type I, lumican and matrix metalloproteinase 12 (MMP12) were evaluated using qRT-PCR and immunocytochemistry respectively.

**Results:**

Cells cultured with AH-enriched FDS media achieved complete wound closure at day 6 post wound creation. The cells cultured in AH-enriched FDS media increased the expression of vimentin, collagen type I and lumican genes and decreased the ALDH, α-SMA and MMP12 gene expressions. Protein expression of ALDH, vimentin and α-SMA were in accordance with the gene expression analyses.

**Conclusion:**

These results demonstrated AH accelerate corneal fibroblasts migration and differentiation of the in vitro corneal ulcer model while increasing the genes and proteins associated with stromal wound healing.

## Background

Corneal wound healing is an exceptionally complex and dynamic process, which is regulated by various growth factors, cytokines, extracellular matrix components and involves intercommunication of various cells [1]. Keratocytes, the quiescent cell type located in the stromal layer, are responsible for generation of the extracellular matrix and maintenance of the shape and transparency of the cornea [2].

Corneal ulcer, either due to traumatic or infective aetiologies, causes disruption of the epithelial layer or may extend deep into the stroma. Stromal reaction upon injury involves apoptosis of the adjacent keratocytes at the wound area while more distant keratocytes become activated to transform into repair phenotypes, the corneal fibroblasts, to reconstruct the damaged stroma and later into myofibroblasts which facilitate wound closure [3]. Corneal fibroblasts then migrate into the wound area triggered by cytokines such as bFGF (basic fibroblast growth factor), insulin-like growth factor 2 and TGFβ1 (transforming growth factor β1) produced by epithelial cells, stromal cells, inflammatory cells, and lacrimal gland cells [1]. The healing process also involves the restoration of corneal transparency, production, deposition and degradation of extracellular matrix components, and scar remodeling [4]. Migration and phenotypical transition of quiescent keratocytes to active phenotypes; corneal fibroblasts and myofibroblasts are the key processes in stromal wound healing.

One of the major concerns in the usage of topical antibiotic or antifungal eye drop in treating corneal injury is the development of resistance and the presence of preservatives that can cause various side effects such as corneal epithelial damage and conjunctival inflammation [5, 6]. Hence, there is a need to search for an alternative or adjunct treatment for corneal injury. Many researchers have directed their focus on the potentiality of natural products in treating various diseases and trying to alleviate the drawbacks of the conventional therapy.

Honey, a carbohydrate-rich natural product originated from floral nectars, has been reported to possess antibacterial, anti-inflammatory and antioxidant properties [7]. Acacia honey (AH) is a monofloral honey produced by *Apis mellifera* honeybees from *Acacia mangium* trees and flowers. It has been documented to accelerate wound contraction in rats, increasing skin-breaking strength and promotes tissue granulation [8]. We hypothesize that AH may exert the same favourable effects on the stromal derived corneal fibroblasts as it has demonstrated on the dermal fibroblasts.

This study aimed to investigate the effects of AH in migration and differentiation during wound healing of corneal fibroblasts. For this purpose, we developed an in vitro corneal ulcer model using 4 mm corneal trephine onto confluent rabbit corneal fibroblasts culture. We hypothesized that supplementation of AH in the culture media promotes the centripetal migration and accelerates healing of the corneal fibroblasts in this ulcer model.

## Methods

This study was performed following approval from the Research and Ethical Committee of Faculty of Medicine, Universiti Kebangsaan Malaysia (UKM project code: GGPM-2011-085) and Universiti Kebangsaan Malaysia Animal Ethics Committee (project code: UKMAEC Approval Number FP/ANAT/2012/ NORZANA/ 18-JANUARY/ 419-JANUARY-2011-DECEMBER-2013-AR-CAT2).

### Acacia honey (AH)

Acacia honey (AH) was purchased from Ministry of Agriculture, Malaysia and gamma irradiated at 25 kGy at Ministry of Science, Technology and Innovation, Malaysia. Our previous study revealed that the optimal concentration of AH was 0.025 % [9]. The same study observed that the AH was not cytotoxic at the range of 0–3.125 % concentrations using H_2_O_2_ at the concentration of 1.56nM as positive control [9].

### Isolation and culture of rabbit stromal derived corneal fibroblasts

Primary cultures of corneal stromal cells were isolated and cultured as described previously [10]. Briefly, six New Zealand white strain rabbits’ corneas (2.0–2.5 kg) were excised by circular incision 2 mm beyond the corneoscleral junction. Connective tissue such as endothelium, ocular muscle, sclera, ciliary body and iris were stripped off and discarded. Following rinsing with phosphate buffered solution (Gibco Invitrogen, USA), corneas were immersed in Dispase solution 2 mg/ml (Sigma-Aldrich, USA) at 4 °C for 18 h to dissociate the stromal layer from the epithelium. Loose epithelial layer were gently removed with a scalpel (Fisher Scientific, Los Angeles, CA) and the remaining stroma was rinsed, sliced and digested with 0.3 % collagenase type I with intermittent gentle shaking at 37 °C for 90 min. The digested stroma was centrifuged at 500 x g for 10 min and the resultant pellet was washed twice with phosphate buffered solution (PBS, pH 7.2, Gibco Invitrogen, USA) to remove the remaining enzyme. Viable stromal cells were seeded in T75 cm^2^ culture flask (BD Falcon, Franklin Lakes, NJ) with seeding density of 5 × 10^4^ cell/cm^2^ in medium containing serum (FDS) consisting of Ham’s F-12: Dulbecco’s Modified Eagle’s Medium (Gibco), with 10 % foetal bovine serum (FBS; Gibco), 1 % of antibiotic and antimitotic (Gibco), 1 % of 50 μg/ml ascorbic acid (Sigma, St. Louis, USA). Stromal derived corneal fibroblasts were cultured in a 5 % CO_2_ incubator (Jouan, Duguay Trouin, SH) under 95 % humidity at 37 °C. At 80 % confluence, the primary culture (P0) was trypsinized using 0.125 % trypsin-EDTA (Gibco) and subcultured to passage 1 (P1) in FDS with seeding density of 5 × 10^3^ cells/cm^2^ in six well-plates (BD Falcon, Franklin Lakes, NJ) under the same condition as the primary culture. Medium was changed every 2 days and the cultures were monitored everyday using inverted phase contrast microscope (Carl Zeiss, Germany) to examine the morphological changes or any sign of contamination.

### Cell migration study and in vitro corneal ulcer healing model

**Fig. 1:**
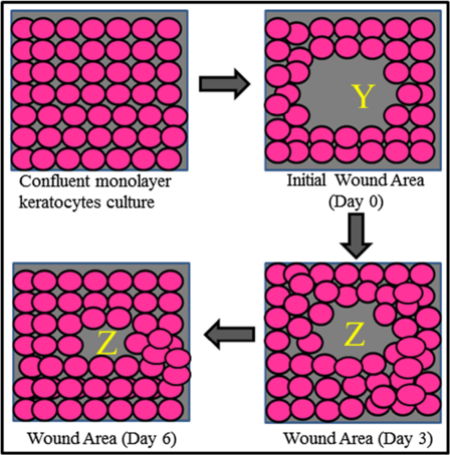
Schematic diagram of in vitro corneal ulcer model using 4 mm corneal trephine onto confluent monolayer corneal fibroblasts at P1. Y represents the wound area at the initial day of experiment (Day 0) and Z represents the wound area at day 3 or day 6 post wound creation

To evaluate the migration of corneal fibroblasts, a defect was created onto confluent monolayer culture. Corneal fibroblasts at passage 1 were seeded in the six well plates (BD Falcon, Franklin Lakes, NJ) at the density of 5 × 10^3^ cells/cm^2^ in FDS medium until it reached confluence at day 3. The medium was discarded and corneal ulcer was created using 4 mm corneal trephine onto the confluent monolayer corneal fibroblasts culture. The cells within the circumference of the wound were gently scraped off using a sterilized cotton bud. The cultures were rinsed twice with PBS after wounding to remove non-adherent cells and cultured in four different media; basal medium (FD), medium containing serum (FDS), FD with supplementation of 0.025 % AH (AH-enriched FD medium), and FDS with supplementation of 0.025 % AH (AH-enriched FDS medium). Cells were cultured at 37 °C in a 5 % CO_2_ incubator and media were changed every 3 days. The wound area was measured using Axiovision LE64 software and the percentage of wound closure was quantified by measuring the average percentage of wound closure (*n* = 6 independent experiments) by migrating cells at the wound edges in the initial day of wounding (day 0), day 3 and day 6 post wound creation (Fig. 1) using the formula below:

Formula:

$$ \mathrm{Wound}\ \mathrm{closure} = \kern0.87em \frac{\mathrm{Y}-\mathrm{Z}}{\mathrm{Y}} \times 100\% $$

$$ Y = \mathrm{Initial}\ \mathrm{wound}\ \mathrm{area} $$

$$ Z = \mathrm{Wound}\ \mathrm{area}\ \mathrm{at}\ \mathrm{day}\ 3\ \mathrm{or}\ \mathrm{day}\ 6 $$

Morphological assessment, gene expression and immunocytochemistry analyses were performed at the same time point of the cell migration study.

### Gene expression of wound healing markers

Total RNA from corneal fibroblasts cultured in four different media at day 0, day 3 and day 6 following in vitro wound creations were extracted using TRI Reagent (Molecular Research Centre, Cincinnati, USA) according to the manufacturer’s protocol. In brief, chloroform was added into the homogenate to isolate the transparent aqueous contained total RNA. Isopropanol and Polyacryl carrier (Molecular Research Centre) were added to each extraction to precipitate the total RNA. The extracted RNA pellet was washed with 75 % ethanol and air dried before dissolving it in Rnase and Dnase free distilled water (Invitrogen, Carlsbad, USA). Complementary DNA was synthesized from 100 ng of Total RNA with Super Script™ III First-Strand Synthesis Super Mix reverse transcriptase (Invitrogen, Carlsbad, USA). The protocol conditions were 10 min at 23 °C for primer annealing, 60 min at 50 °C for reverse transcription and 5 min at 85 °C for reaction termination. The expression of aldehyde dehydrogenase (ALDH), vimentin, alpha-smooth muscle actin (α-SMA), collagen type I, lumican and matrix metalloproteinase 12 (MMP12) were evaluated by two-step reverse transcriptase-polymerase chain reaction (Invitrogen, Carlsbad, USA). Positive control was observed using GAPDH primer pairs. All gene-specific primers used are shown in Table 1. The two-step RT-PCR reaction was performed using SYBR Green as the indicator in a Bio-Rad iCycler (Bio-Rad, USA). RT-PCR was carried out using 12.5 μl of iQ SYBR Supermix, 1 μl each of forward and reverse primers (GenBank, using Primer-3), 8.5 μl deionized water and 1 μl of cDNA template. The cycling conditions for all primer pairs were as follows: cycle 1: 95 °C for 3 min (1×), cycle 2: Step 1 95 °C for 10 s and Step 2 61 °C for 30 s (40×), followed by melting curve analysis. The specificity and PCR product size were visualized by 2 % agarose gel electrophoresis with ethidium bromide staining.

**Table 1:** Description of primers used in qRT-PCR

Primer	Accession Number	Primer Sequence	Product size
GADPH	NM_001082253	F:caa cga att tgg cta cag ca	186
		R:aaa ctg tga aga ggg gca ga	
ALDH	AY508694	F:gag tgg cat gat tca gtg agc	186
		R:gag tag tcg tcc cct ctt gga	
Vimentin	AY465353.1	F: tgc agg aag aga ttg cct tt	117
		R: tga ggt cag gct tgg aga ca	
α-SMA	X60732	F: tcg aca tca gga agg acc tct	206
		R: cat ctg ctg aaa ggt gga cag	
Lumican	AF020292	F: ctg cag ctt acc cac aac aag	160
		R: ggt tga agc tca agt cca ggt	
Collagen type I	AY633633	F: gcg gag agt act gga ttg acc	163
		R: cac acg tgc ttc ttc tcc ttg	
MMP12	AB006779	F: tga agc gtg agg atg ttg ag	181
		R: aaa gca tgg gct atg aca cc	

### Immunocytochemistry

Corneal fibroblasts cultured on cover glass in four different media at day 0, day 3 and day 6 post wound creation were fixed with 4 % paraformaldehyde at 4 °C using standard protocol from Dako Animal Research Kit. Briefly, the slides were rinsed with running tap water for 3 min. Following incubation with blocking agent (0.03 % peroxidase block) at room temperature for 5 min, cells were labelled using the Biotinylation reagent and incubated with primary antibodies for 30 min. Primary antibodies used were anti-ALDH (1:100, Dako), anti-Vimentin (1:200, Dako) and anti-α-SMA (1:100, Dako). Biotinylation reagent was mixed to bind biotinylated secondary antibody to the primary antibody. The specimen was then incubated with streptavidin-peroxidase, followed by reaction with diaminobenzidine/hydrogen peroxide as substrate-chromogen. Nuclei were stained with haematoxylin (Sigma). Positive stained cells exhibited brownish precipitate in the cytoplasm. Coverslips were mounted using DPX mounting medium (Sigma Aldrich Co, USA) and slides were observed using confocal laser scanning microscope (LSM-510, Zeiss).

### Data analysis

Values were expressed as mean ± standard error of mean (SEM). The data was analyzed with paired *t*-test and ANOVA using SPSS version 20.0 and the p value of <0.05 was considered to be significant.

## Results

### Morphology of the corneal fibroblasts at the wound edge

**Fig. 2:**
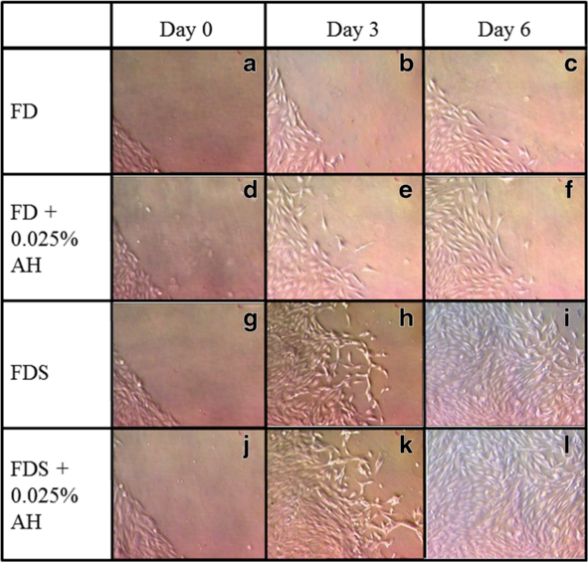
Phase contrast micrograph representing one ninth of the whole wound edge of the corneal fibroblasts (P1) cultured in four different media; 1) FD medium (**a**, **b**, **c**), 2) AH-enriched FD medium (**d**, **e**, **f**), 3) FDS medium (**g**, **h**, **i**), and 4) AH-enriched FDS medium (**j**, **k**, **l**). Spindle-shaped cells resembling active fibroblasts are shown by arrows. The wound area was observed at day 0, day 3 and day 6 post wound creation. Magnification ×20

Corneal fibroblasts underwent centripetal migration within 3 h post wound creation. Cells at the wound edge changed morphology to become spindle-shaped cells resembling active fibroblast (Fig. 2).

### Cell migration study and in vitro corneal ulcer healing model

**Fig. 3:**
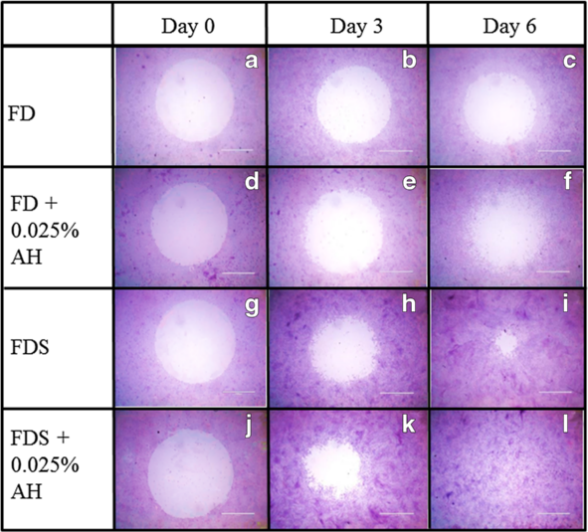
Micrographs showed the wound area of cultured corneal fibroblasts stained with H & E at passage 1 in four different media; 1) FD medium (**a**, **b**, **c**), 2) AH-enriched FD medium (**d**, **e**, **f**), 3) FDS medium (**g**, **h**, **i**), and 4) AH-enriched FDS medium (**j**, **k**, **l**). The wound area was observed at day 0, day 3 and day 6 post wound creation. Magnification ×20

**Fig. 4:**
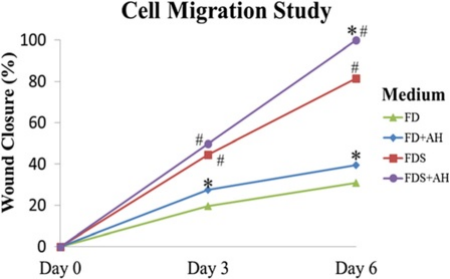
Cell migration study of corneal fibroblasts cultured in four different media of the in vitro wound healing model (*n* = 6 independent experiments). (*) denotes significant difference (*p* < 0.05) between the same medium while (#) denotes significant difference (*p* < 0.05) between different media

Cell migration study signifies the centripetal migration of the corneal fibroblast in closing the wound. The cells cultured in AH-enriched FD and FDS media showed higher percentage of wound closure compared to their respective controls (Fig. 3). At day 3, cells cultured in AH-enriched FD medium showed a 27.4 % reduction in the wound size compared to 19.5 % for cells cultured in FD medium alone (Fig. 3b, e and 4). Cells cultured in AH-enriched FDS medium reduced the wound area to 49.9 % compared to that of 44.5 % for cells cultured in FDS medium alone (Fig. 3h, k and 4). At day 6, the migration of cells were significantly slower in FD medium compared to AH-enriched FD medium which showed 30.8 and 39.5 % wound closure respectively (Fig. 3c, f and 4). Cells cultured in AH-enriched FDS medium showed complete wound closure at day 6 (Fig. 3l), compared to only 81.5 % wound closure in the FDS medium alone (Fig. 3i and 4). These results indicate that AH at the concentration of 0.025 % has significant effect in accelerating the migration of the cultured corneal fibroblasts of the in vitro corneal ulcer model.

### Gene expression of the differentiation and wound healing markers

**Fig. 5:**
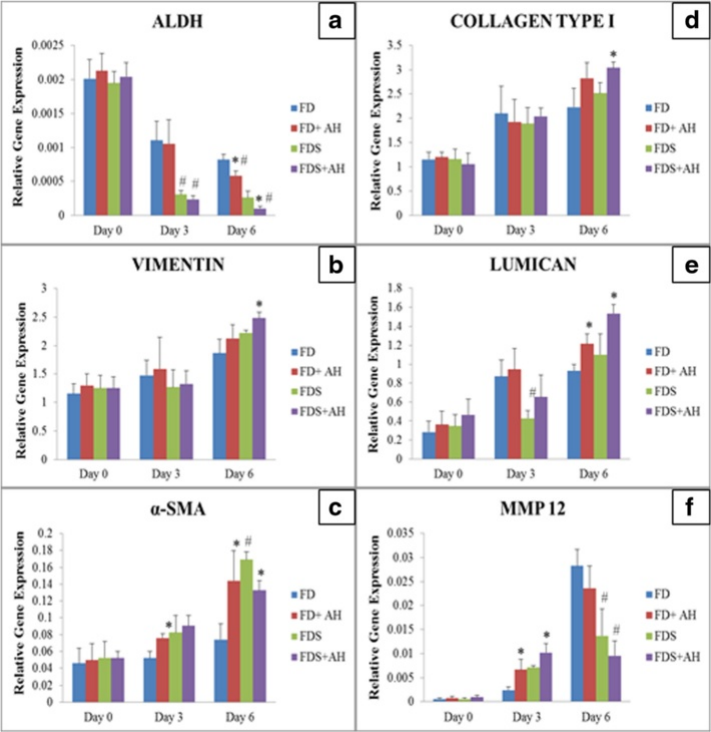
Gene expression of corneal fibroblasts differentiation and wound healing markers; **a** ALDH, **b** Vimentin, **c** α-SMA, **d** collagen type I, **e** lumican and **f** MMP12 for corneal fibroblasts cultured in four different media. Values were expressed as mean ± SEM, *n* = 6 independent experiments. (*) denotes significant difference (*p* < 0.05) between the same medium (#) denotes significant difference (*p* < 0.05) between different media

The differentiation and wound healing markers were evaluated by gene expression study. The expression of ALDH was the highest at the initial day of wound creation in all groups (Fig. 5a). At day 3, the expression of ALDH reduced significantly in FDS and AH-enriched FDS media compared to FD and AH-enriched FD media, respectively. At day 6, corneal fibroblasts cultured in AH-enriched media significantly reduced the expression of ALDH compared to their respective controls. In contrast, vimentin showed increasing expression pattern from the initial day of wound creation until day 6 in all groups (Fig. 5b). Cells cultured in AH-enriched FDS media showed the highest vimentin expression compared to FDS medium alone.

Cells showed increasing trend of α-SMA expression, compared to the initial day of wounding in all groups (Fig. 5c). At day 6, corneal fibroblasts cultured in FDS medium showed higher expression of α-SMA compared to FD medium. However, the expression of α-SMA was significantly reduced in AH-enriched FDS media. The expression of collagen type I gene was up-regulated at day 3 and day 6 in comparison to the initial day of wound creation in all groups (Fig. 5d). Corneal fibroblasts cultured in AH-enriched FDS medium showed the highest expression of collagen type I compared to control medium at day 6 of wound creation.

The expression of lumican was up-regulated for cells cultured in all media (Fig. 5e). At day 6, expression of lumican increased significantly (*p* < 0.05) when corneal fibroblasts were cultured in AH-enriched media compared to their respective controls. Gene expression of MMP12 was the lowest at the initial day of wound creation in all media (Fig. 5f). At day 3, MMP12 gene expression was up-regulated, especially in cells cultured in AH-enriched FDS medium compared to FDS medium alone. At day 6, corneal fibroblasts cultured in FDS groups showed reduction in the gene expression of MMP12 compared to the FD groups.

**Fig. 6:**
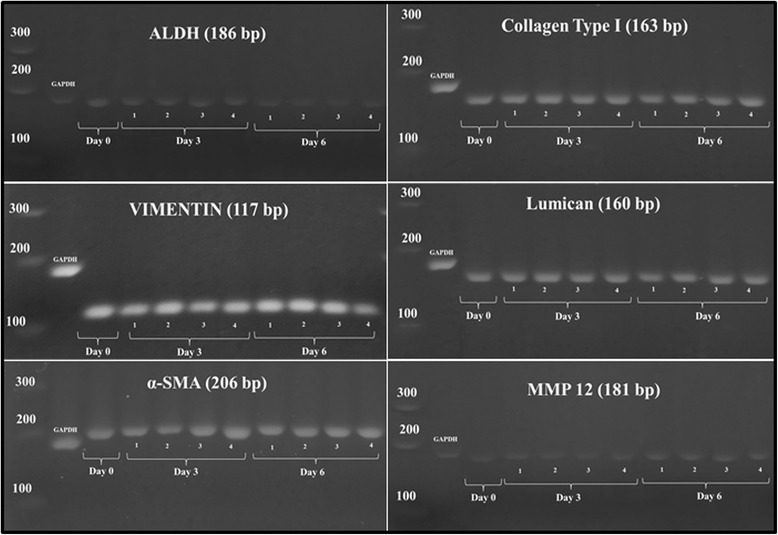
Gel electrophoresis for confirmation of product size of cultured corneal fibroblasts in four different media; (1) FD, (2) AH-enriched FD medium, (3) FDS and (4) AH-enriched FDS medium. GADPH gene was used as internal control

Gel electrophoresis showed specific product size with the presence of single band for gene expression of ALDH, vimentin, α-SMA, collagen type I, lumican and MMP12 in corneal fibroblasts cultured with or without supplementation of 0.025 % AH (Fig. 6).

### Immunocytochemistry

**Fig. 7:**
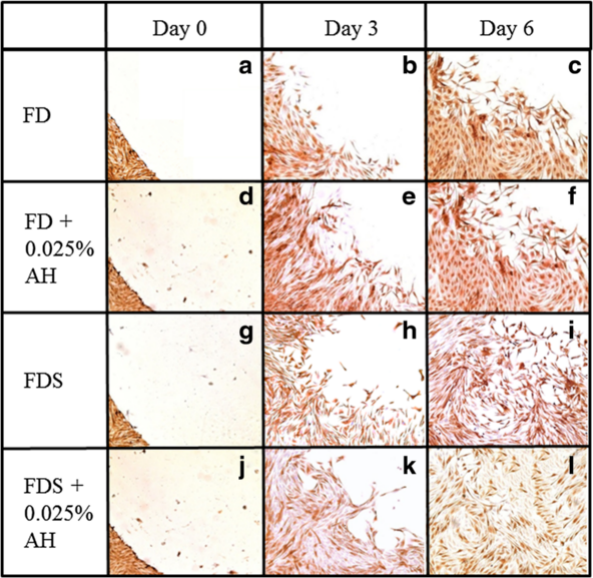
Immunocytochemistry staining for ALDH protein of corneal fibroblasts cultured in four different media; 1) FD medium (**a**, **b**, **c**), 2) AH-enriched FD medium (**d**, **e**, **f**), 3) FDS medium (**g**, **h**, **i**), and 4) AH-enriched FDS medium (**j**, **k**, **l**) at day 0, day 3 and day 6 post wound creation. Magnification ×50

The protein expression during wound healing was ascertained through immunohistochemistry. Abundant positive brown stained cells for ALDH protein was detected at the wound edge at the initial day of wounding in all groups (Fig. 7a, d, g, j). The protein expression was reduced during day 3 (Fig. 7b, e h, k) and day 6 in AH-enriched media compared to their respective controls (Fig. 7c, f, i, l).

**Fig. 8:**
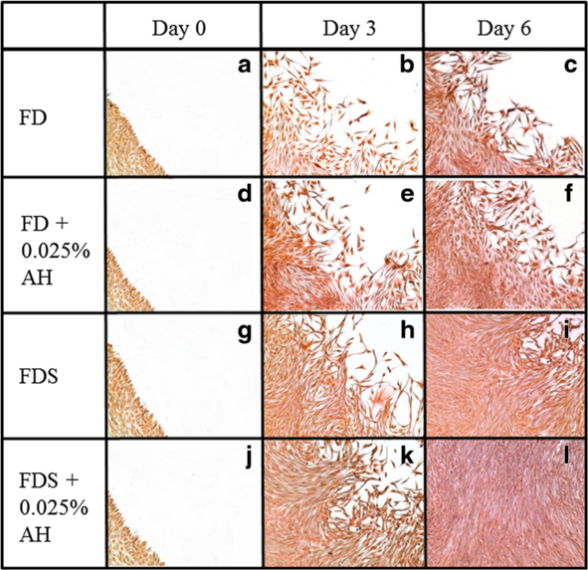
Immunocytochemistry staining for vimentin protein of corneal fibroblasts cultured in four different media; 1) FD medium (**a**, **b**, **c**), 2) AH-enriched FD medium (**d**, **e**, **f**), 3) FDS medium (**g**, **h**, **i**), and 4) AH-enriched FDS medium (**j**, **k**, **l**) at day 1, day 3 and day 6 post wound creation. Magnification ×50

Vimentin was highly expressed throughout the study in all culture media (Fig. 8). The density of positive stained cells was increased in cell cultured in FD (Fig. 8b, e, c, f) and FDS (Fig. 8h, k i, l) media supplemented with 0.025 % AH compared to control media at day 3 and day 6 post wounding.

**Fig. 9:**
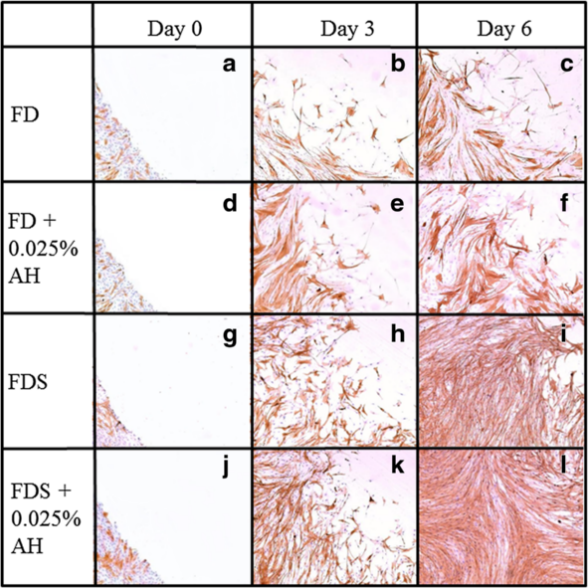
Immunocytochemistry staining for α-SMA protein of corneal fibroblasts cultured in four different media; 1) FD medium (**a**, **b**, **c**), 2) AH-enriched FD medium (**d**, **e**, **f**), 3) FDS medium (**g**, **h**, **i**), and 4) AH-enriched FDS medium (**j**, **k**, **l**) at day 1, day 3 and day 6 post wound creation. Magnification ×50

At the initial day of wounding, α-SMA protein expression was reduced in all groups (Fig. 9a, d, g, j). At day 3 and day 6, α-SMA protein expression were increased especially in corneal fibroblasts cultured in AH-enriched media (Fig. 9e, f, k, l) compared to controls, respectively (Fig. 9b, c h, i), indicating the presence of myofibroblasts phenotype in the wound. All the immunocytochemistry results were in agreement with the gene expression analyses.

## Discussion

The process of corneal stromal healing involved the activation of quiescent keratocytes and transition into repair phenotypes; the corneal fibroblasts and myofibroblasts. These repair phenotypes are important to stimulate the regeneration of the corneal tissue during stromal wound healing [11]. Corneal fibroblasts migrate and synthesize new extracellular matrix, while myofibroblasts are essential for wound contraction during healing [12, 13].

The present study showed corneal fibroblasts in AH-enriched media promotes faster migration and wound closure compared to controls, respectively. This is due to the presence of glucose in AH that provides main source of energy for cell proliferation and growth [14]. Previous study from this group showed AH increased the proliferative capacity of corneal fibroblasts in vitro [9].

Glucose provides energy during angiogenesis and deposition of new tissue [15]. AH has been reported to possess the highest sugar content compared to other honeys in Malaysia and this provides metabolic support for the migration and proliferation of corneal fibroblasts in corneal wound healing [16]. Previous study revealed AH enhanced granulation of the skin of the rats with burn injury [8]. Similar results were reported in other in vivo researches using Gelam honey, Chestnut honey, Blossom honey and Phododendron honey on rats’ skin wound healing [17, 18].

Hydrogen peroxide is a strong antibacterial agent for *Escherichia coli* and *Pseudomonas aeruginosa* in vitro [19]. Bang et al. (2003) reported hydrogen peroxide was produced at low concentration and in a continuous manner when honey was diluted [20]. Previous study demonstrated that low concentration of hydrogen peroxide ranging from 10 nM to 1 μM was sufficient to stimulate the migration of hamster and rat fibroblasts in vivo after injury [21]. Hydrogen peroxide provided adequate oxygen and nutrient to the injured tissue through angiogenesis [22]. This antibacterial function of hydrogen peroxide in the AH provides a favourable and optimal culture condition for the migration and proliferation of corneal fibroblasts during wound closure.

Chronic wounds have a raised alkaline environment. Honey has been reported to lessen wound pH, reduce protease activity, increase fibroblasts activity and elevate oxygen release which in turn facilitates in wound healing [23]. Other research demonstrated that acidic pH promotes wound contraction by myofibroblasts in the skin [24]. Hence, AH with the pH of 3.53 was optimal for corneal fibroblasts migration in wound healing [16].

The healing process of the corneal stroma starts with the initiation of neighbouring keratocytes migrating to the acellular region at the edge of the wound [2]. Keratocytes increase in size and number, leading to reduction of expression of corneal crystallins such as ALDH and loss of cellular transparency [25]. ALDH expression was down-regulated significantly in AH-enriched FDS medium. The presence of serum or growth factors stimulated transition of quiescent keratocytes into corneal fibroblasts and myofibroblasts phenotypes which in turn lowered the expression of ALDH [26]. The repair phenotypes were not reversible to become quiescent keratocytes even with subsequent culture of the corneal fibroblasts in the serum-free medium [27]. Immunocytochemistry finding of ALDH protein was in accordance to the gene expression results.

During healing, spindle-shaped corneal fibroblasts migrated into the acellular region within 24 h and synthesized extracellular matrix [2]. The expression of stromal proteins such as vimentin, decorin and keratocan was reported to increase following keratoconus surgery [28]. Growth factors such as fibroblast growth factor-2 (FGF-2) and platelet-derived growth factor (PDGF) in the serum or in the culture medium initiate the stromal keratocytes to differentiate into corneal fibroblasts or myofibroblasts comparable to the keratocytes reaction to injury in vivo [29]. Immunocytochemistry staining for vimentin was increased 1 week following photorefractive keratectomy in the rabbit cornea and 2 weeks following cornea alkaline abrasion in rabbit [30, 31]. Corneal fibroblasts cultured in AH-enriched FDS media expressed the highest vimentin level indicating the proliferation during wound closure. Application of AH onto injured skin was reported to promote wound contraction by dermal fibroblasts [32].

Myofibroblasts, which are characterized by the presence of α-smooth muscle actin (α-SMA), appeared during the contraction phase of corneal wound healing [33]. Myofibroblasts differentiation is associated with dilution of corneal crystallins and reduction of mRNA levels for ALDH [13]. Our study showed an increasing trend of α-SMA expression for corneal fibroblasts treated with AH-enriched media which indicates that AH induced the differentiation of corneal fibroblasts to myofibroblasts during healing. Jester et al. (1995) reported a direct association between the expressions of α-SMA with the stages of corneal wound contraction [34]. Disappearance of myofibroblasts during complete wound closure was mediated by apoptosis and differentiation of myofibroblasts back to fibroblasts during stromal re-remodeling leading to regression of corneal haze [35]. Similarly, our study showed the reduction in the α-SMA expression during wound closure in the AH-enriched FDS medium and this favourable finding indicates the potential of AH in reducing corneal opacity and scarring.

During stromal wound healing, the mRNA expression of collagen type I and V were increased in both fibroblasts and myofibroblasts [26, 31]. The up-regulation of collagen type I in AH-enriched media was in agreement to the findings of the full-thickness wound injury in the rat treated with honeybee venom [36].

Lumican is expressed by quiescent keratocytes in normal unwounded cornea and plays an important role in corneal transparency through the regulation of collagen matrix assembly [4]. The increase expression of lumican in the AH-enriched media indicates the potential role of AH in promoting corneal transparency during corneal wound healing.

Matrix metalloproteinase-12 (MMP12) is essential in tissue remodelling during corneal wound healing [37]. Following corneal injury, a glycoprotein called extracellular matrix metalloproteinase inducer is produced on the surface of corneal epithelial cells which interact with corneal fibroblasts to produce MMP and induce myofibroblasts differentiation [38]. Keratocytes and fibroblasts expressed abundant MMP12 only during the acute phase but reduced during final stages of wound healing as transforming growth factor-β1 (TGF-β1) induced the differentiation of fibroblasts to myofibroblasts [39]. The presence of TGF-β1 in the serum may explain the down-regulation of MMP12 in corneal fibroblasts cultured in FDS medium with or without supplementation of AH.

There were few limitations in the study. The mechanism of action of AH could be well explained if a mixture of carbohydrates such as glucose and fructose, similar to that of AH composition, is included as a control. This aspect would be dealt in detail in future studies to elucidate the mechanism of wound healing by AH. We did not use any drug as positive control because the ulcer created was not due to any infective cause.

## Conclusion

In conclusion, the supplementation of Acacia honey into the culture media accelerates rabbit corneal fibroblasts migration and differentiation of the in vitro corneal wound healing model. The close similarity of the anatomical structure of rabbit cornea to the human cornea serves as a basis for the relevance of the above findings. This provides an opportunity for the development of honey-based eye drop for therapeutic purposes or as an adjunct in treating corneal injury in the future.

## Abbreviations

AH, acacia honey; ALDH, aldehyde dehydrogenase; bFGF, basic fibroblast growth factor; FBS, foetal bovine serum; FD, basal medium; FDS, medium containing serum; MMP12, matrix metalloproteinase 12; P1, passage 1; qRT-PCR, real time quantitative reverse transcription polymerase chain reaction; SEM, standard error of mean; TGFβ1, transforming growth factor β1; α-SMA, alpha-smooth muscle actin
